# The case of late diagnosis of giant parathyroid adenoma in combination with fibrocystic osteitis and brown tumor of the upper jaw: a case report

**DOI:** 10.14341/probl12713

**Published:** 2021-03-14

**Authors:** G. A. Bersenev, E. A. Ilyicheva, E. G. Griroryev

**Affiliations:** Irkutsk Scientific Centre of Surgery and Traumatology; Irkutsk Scientific Centre of Surgery and Traumatology; Irkutsk Scientific Centre of Surgery and Traumatology; Irkutsk State Medical University

**Keywords:** primary hyperparathyroidism, giant parathyroid adenoma, fibrocystic osteitis, brown tumor, surgical treatment, parathyroid adenomectomy, case report

## Abstract

In this case report the authors inform about late diagnosis of giant adenoma of the parathyroid gland with primary hyperparathyroidism (PHPT) and the development of fibrocystic osteitis with a «brown» tumor of the upper jaw. The patient has been under the care endocrinologist with type 2 diabetes mellitus and multinodular goiter for 8 years.The last 5 years there was a clinical manifestation of PHPT, but the diagnosis was made by an oncologist after the detection of a «brown» tumor of the upper jaw. According to multispiral computed tomography and scintigraphy with 99mTc-MIBI, a focal lesion was found in the upper jaw on the right, lytic foci in the bones of the cranial vault, pelvis, lower extremities, ribs on the right, as well as a giant parathyroid adenoma on the right. According to the increased risk of the patient having a malignant neoplasm of the parathyroid gland, an extended surgical treatment of PHPT in the enblock volume was carried out with the achievement of remission of the PHPT. This clinical case illustrates a variant of the severe course of PHPT with the development of such a rare complication as fibrocystic osteitis and demonstrates the importance of timely diagnosis.

## Abstract (ru)

Обсуждается поздняя диагностика гигантской аденомы околощитовидной железы (ОЩЖ) с первичным гиперпаратиреозом (ПГПТ) и развитием фиброзно-кистозного остеита с «бурой» опухолью верхней челюсти. В течение 8 лет пациентка наблюдалась у эндокринолога по месту жительства по поводу сахарного диабета 2 типа и многоузлового зоба.Последние 5 лет присутствовала клиническая картина ПГПТ, однако диагноз был установлен онкологом после выявления «бурой» опухоли верхней челюсти. По данным мультиспиральной компьютерной томографии и сцинтиграфии с 99mTc-МИБИ обнаружены очаговое образование в верхней челюсти справа, литические очаги в костях свода черепа, таза, нижних конечностей, ребрах справа, а также гигантская аденома ОЩЖ справа. Учитывая повышенный риск наличия у пациентки злокачественного новообразования ОЩЖ, проведено расширенное хирургическое лечение ПГПТ в объеме enblock с достижением ремиссии ПГПТ. Данное клиническое наблюдение иллюстрирует вариант тяжелого течения ПГПТ с развитием такого редкого осложнения, как фиброзно-кистозный остеит, и демонстрирует важность своевременной диагностики.

## BACKGROUND

According to the Russian register of patients with primary hyperparathyroidism (PHPT), the detection rate of this disease in the Russian Federation is 1.3 per 100 thousand population [1]. In 80-90% of cases, sporadic PHPT is caused by adenoma of the single parathyroid gland (PTG), in 10–15% — hyperplasia of four PTGs, in 5% - multiple adenomas and less than 1% — PTG cancer [2]. Giant parathyroid adenoma (weight is more than 3.5 g) is rare pathology. Some reports describe the weight of the tumor from 110 to 145 g [3, 4]. It is the cause of the PHPT development with a high blood level of parathyroid hormone and severe hypercalcemia [5].

Fibrocystic osteitis is a rare and severe complication of PHPT which is manifested by pathological fractures, skeletal deformation with development of cysts (brown tumors) [6]. The latter are found uncommonly in PHPT (4.5%) [7], and are extremely rare as the onset of the disease [8, 9].

We present clinical case of the patient with PHPT with a giant parathyroid adenoma was diagnosed after revealing of maxilla brown tumor.

## CASE DESCRIPTION

A 56-year-old female patient was observed for 8 years by an endocrinologist at the place of residence for type 2 diabetes mellitus and multinodular goiter. First complaints to the pain in bones and major joints, muscle weakness appeared 5 years ago. No any examination was performed. Over the past six months, she lost 20 kg, the intensity of pain increased that limited independent movement, mood swings (irritability, tearfulness) appeared. In this regard, in December 2019, the patient was examined by an oncologist at the place of residence. Maxilla mass lesion has been found on the right. According to multispiral computed tomography (MSCT) of the skull and cervical spine (January 03, 2020), maxilla tumor was confirmed. The lesion area extends to the maxillary sinuses, ethmoid bone, alveolar process and nasal cavity (Fig. 1). The dystrophy of the bones of skull, cervical spine with a lytic focus in the frontal bone on the right is revealed. MSCT of thoracic and abdominal organs (January 03, 2020) was performed. There was a total osteolytic lesion of the ribs, thoracic spine, chronic cystic calculous pancreatitis with intraductal calculi. In addition, total osteolytic lesion of the lumbar spine was determined. According to MSCT, there are no pathological changes in the kidneys and uterus. According to the results of the needle biopsy of maxillary sinus (January 17, 2020), a large number of multinucleated giant cells as well as single oval mononuclear cells without signs of atypia, erythrocytes were revealed.

**Figure:**
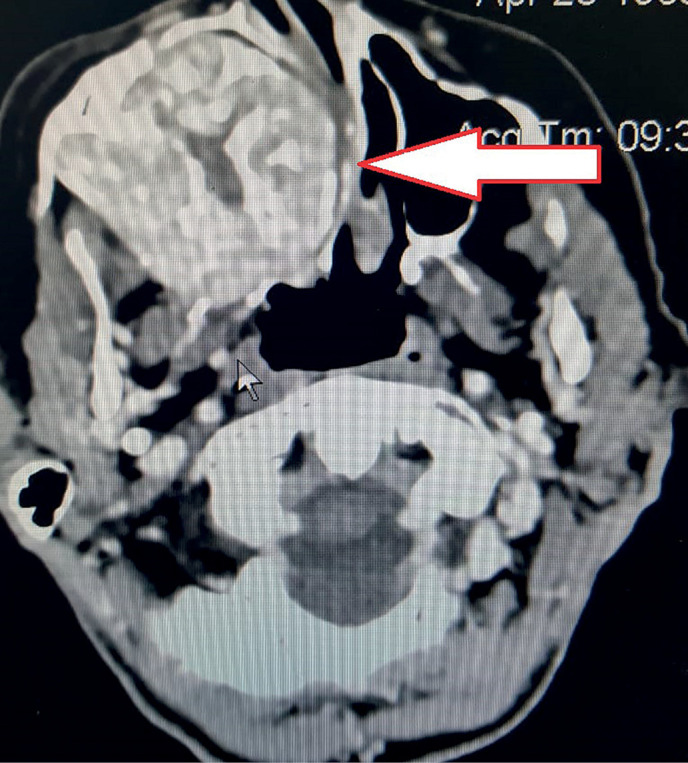
Figure 1. Computed tomography of the facial skull: the arrow shows the mass lesion of the maxillary sinus with spreading to the sphenoid sinus.

In January 2020, intact parathyroid hormone (IPTH) was tested for the first time, the level of which was 2285.1 pg/ml. The patient’s family history is not burdened. Consultation by endocrinologist is recommended.

Results of physical examination, laboratory tests and instrumental investigations

On examination, the patient is cachectic: height is 166 cm, weight is 40 kg, body mass index is 14.5 kg/m2. She moves with the help of a cane and accompanied by a relative. The skin is pale pink; the subcutaneous fat is poorly developed. The thyroid gland (TG) is not enlarged. A solid, painless lesion up to 4 cm is palpable in the projection of the right lobe. The respiratory rate is 16 per minute. In the lungs, breathing is vesicular; no rales are heard. Heart sounds are muffled, rhythmic. Heart rate and pulse rate is 97 per minute. Blood pressure on both brachial arteries is 160/80 mmHg. The results of laboratory test dated February 20, 2020 are presented in the table.

**Table:** Table. Laboratory test results

Thyroid stimulating hormone (TSH)	0.41	µIU/ml	0.4-4.00
Free thyroxine (free T4)	11.5	pmol/l	9.00-22.20
Vitamin D	5	ng/ml	>30
Intact parathyroid hormone (IPTH)	1931.26	pg/ml	15.0-68.3
Albumin-adjusted calcium	4.12	mmol/l	2.1-2.6
Ionized calcium	1.63	mmol/l	1.15-1.27
Calcitonin	<2	pg/ml	≤9.52
Creatinine	60	µmol/l	50.0-120.0
eGFR by formula СKD-ЕРI (2011)	98	ml/min/1.73 m2	>60

The patient was hospitalized in the Endocrinology Department (February 20, 2020). The daily urinary calcium excretion (February 25, 2020) was 4.16 mmol/day (2.5–6.25). Ultrasound examination (US) of thyroid gland and parathyroid gland (02/21/2020) showed that the volume of the right lobe is 17.8 cm3, of the left lobe is 3.7 cm3, the total volume is 21.5 cm3.

A conglomerate of hypoechoic lesions with calcifications and fluid inclusions with a total size of 45×30×20 mm is determined in the middle and lower third of the right lobe of the thyroid gland. In addition, a homogeneous hypoechoic lesion of 12×8×7 mm in size was found closer to the isthmus in the right lobe of the thyroid gland; a similar lesion of 3×3×2 mm in size was found in the lower third of the left lobe of the thyroid gland. Parathyroid glands were not found in a typical location. The data obtained correspond to the TI-RADS 4 category as modified by J.Y. Kwak et al. (2011). Regional lymph nodes are of 5–7 mm in size, with vascularization; cystic changes, hyperechoic inclusions are absent. A fine needle aspiration biopsy (FNA) was performed. Cytological conclusion for aspirates (February 24, 2020) of all thyroid nodules of > 1 cm in diameter: nodular colloid goiter that corresponds to diagnostic category II according to the Bethesda classification (2017).

MSCT of the neck (February 28, 2020) revealed a mass lesion of the right upper PTG of 46x23x28 mm in size, irregular shape with unclear contours, heterogeneous in structure with calcium inclusions. After intravenous enhancement, the lesion uptakes contrast and the ventral surface intimately adjoins and squeezes the right lobe of the thyroid gland, and the lateral surface is adjacent to the right cervical neurovascular bundle (Fig. 2). No evident invasion of the lesion into nearby tissues was found.

**Figure:**
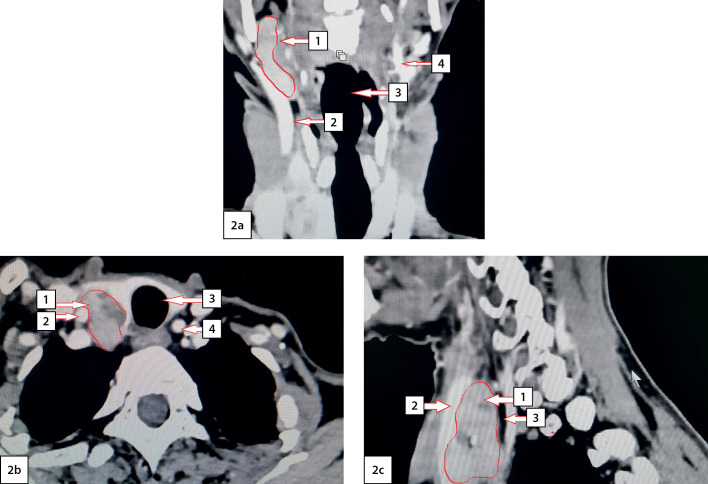
Figure 2. Computed tomography of the neck with angiography: 2a — frontal view; 2b — axial view; 2c — sagittal view. Arrows indicate anatomical structures: 1 — the upper right parathyroid gland; 2 — right common carotid artery; 3 — trachea; 4 — left common carotid artery.

According to scintigraphy of the PTG with 99mTc-technetril in combination with single-photon emission computed tomography (03/02/2020): a focus of radiopharmaceutical (RP) hyperfixation, large, irregular in shape with uneven contours is visualized in the projection of the right lobe of the thyroid gland (Fig. 3).

**Figure:**
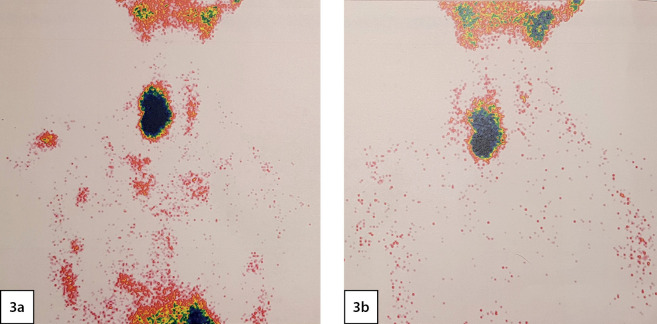
Figure 3. Scintigraphy of the parathyroid glands: 3a - thyroid phase; 3b - parathyroid phase. A focus of radiopharmaceutical (RP) hyperfixation, large, irregular in shape with uneven contours is visualized in the projection of the right lobe of the thyroid gland.

According to the results of bone scintigraphy using RP — 99mTc-pyrfotech (03.03.2020), multiple areas of RP uptake in the bones corresponding to MSCT (skull, thoracic and abdominal organs dated January 03, 2020), of local lytic destruction were revealed. Findings: maxilla focal lesion on the right, increased RP uptake and lytic foci in the bones of the cranial vault, ribs on the right, in the bones of pelvis and lower extremities (Fig. 4). The nature of the skeletal lesion corresponds to the manifestations of fibrocystic osteitis or metastases of PTG cancer.

**Figure:**
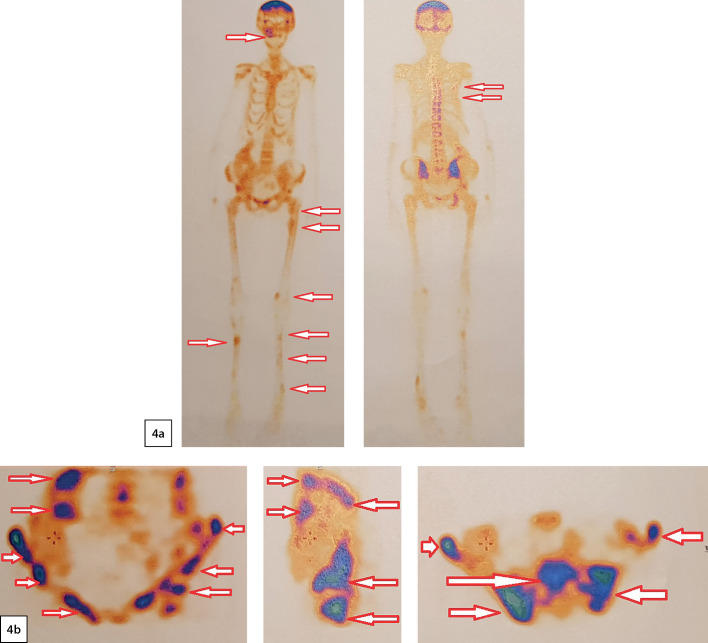
Figure 4. Bone scintigraphy: 4a - scintigraphy of bones in the «whole body» mode: arrows indicate maxilla focal lesion on the right as well as focal lesions in the region of the ribs on the right, lower extremities; 4b - pelvic scintigraphy: arrows indicate multiple lytic lesions in the pelvic bones.

Taking into account the manifestations of life-threatening hypercalcemia with a high risk of hypercalcemic crisis, the manifest form of hyperparathyroidism with severe bone manifestations and the development of fibrocystic osteitis, urgent surgical treatment of PHPT is scheduled. As a preoperative preparation, hypercalcemia was corrected by forced diuresis (0.9% NaCl 2000.0 ml IV + furosemide 60 mg IV for 2 days) and bisphosphonate (zoledronic acid 4 mg + 0.9% NaCl 200.0 ml IV once) which led to decrease in ionized calcium level — 1.51 mmol/l (March 09, 2020). For further treatment on March 10, 2020, the patient was transferred to the Department of Thoracic Surgery.

The surgery was performed on March 11, 2020. An encapsulated right upper thyroid gland of dark brown color, of 6.0x5.0x4.0 cm in size, occupying the entire space between the right non-enlarged lobe of the thyroid gland (intimately connected with it), trachea, esophagus medially and the right neurovascular bundle — laterally was found dorsally to the right recurrent laryngeal nerve (RLN) (Fig. 5). The right lower PTG of 0.6×0.5×0.3 cm in size, of gray-yellow color was revealed ventrally to the right RLN at the level of the lower third of the right lobe of the thyroid gland caudal to the lower pole. Considering that the significantly enlarged right upper thyroid gland is tightly attached to the right lobe of the thyroid gland, the right upper paraadenomectomy and right-sided hemithyroidectomy were performed. Dynamics of IPTH level intraoperative monitoring: before removal of the PTG — 2,500 pg/ml; 10 minutes after removal — 65.2 pg/ml. Intraoperative test by Miami criterion is positive [10]. The total weight of the removed gross preparation was 50 g, the right upper thyroid gland adenoma — 30 g (Fig. 6).

**Figure:**
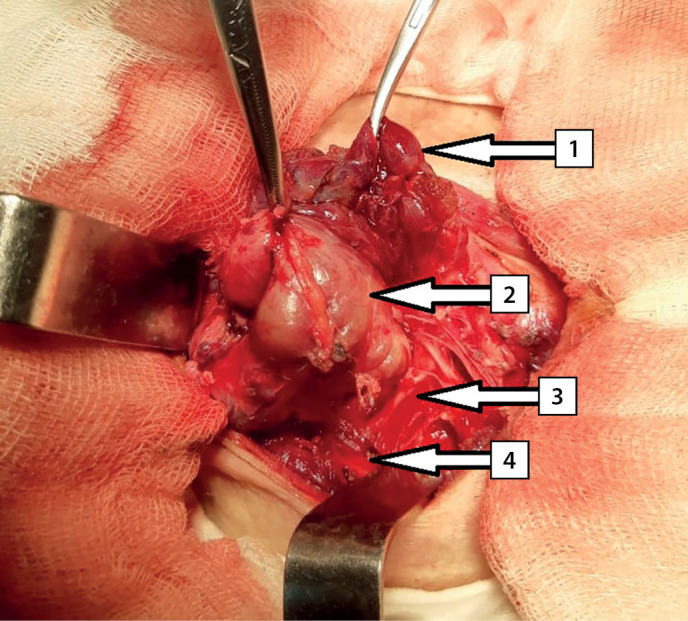
Figure 5. Intraoperative photography. Arrows indicate anatomical structures: 1 — the right lobe of the thyroid gland; 2 — right upper parathyroid gland; 3 — right recurrent laryngeal nerve; 4 — the right common carotid artery.

**Figure:**
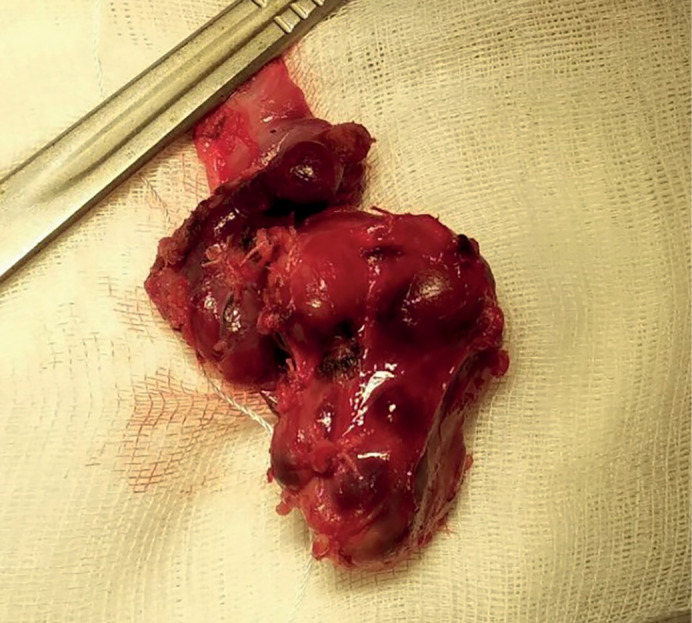
Figure 6. Gross preparation. The right upper parathyroid gland removed as enblock with the right lobe of the thyroid gland.

According to the histological examination, the right upper PTG is represented by an adenoma consisting of active clear cells with single accumulations of oxyphilic cells. The gland had its own capsule and an area of unchanged tissue consisting of inactive clear cells along the periphery (Fig. 7). The right lobe of the thyroid gland is represented by a picture of nodular hyperplasia. The main tissue has a normal follicular structure. The nodes are predominantly of normal follicular structure with moderate proliferation of the thyroid epithelium.

**Figure:**
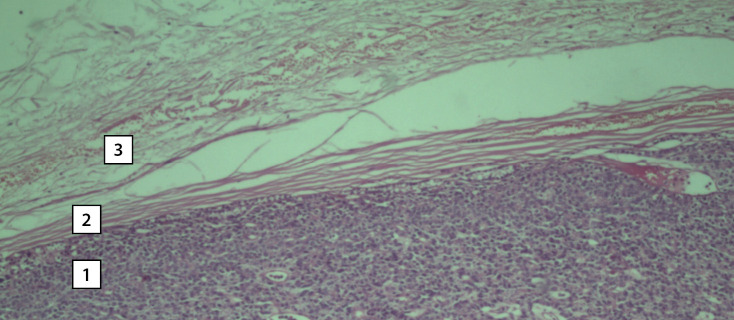
Figure 7. Photomicrograph of the surgical material. Hematoxylin–eosin staining. Magnification 10x0.25. The tissue of the right upper parathyroid gland. A section of adenoma tissue consisting of dark clear cells (1), a section of the connective tissue capsule (2), a section of cells of unchanged tissue with light clear cells (3).

In the postoperative period, laryngoscopy was performed: symmetrical mobility of the vocal folds was established. On the Day 1 after surgery, iPTH level was 26.0 pg/ml, albumin–adjusted calcium level was 1.8 mmol/l, ionized calcium level was 0.63 mmol/l that was accompanied by a clinical picture of hypocalcemia in the form of ossalgia. Prescribed intravenous infusion of calcium gluconate solution 100 mg/ml — 30.0 ml 3 times a day as well as calcium carbonate tablets 8 g per day and alpha–calcidol capsules 4 μg per day that allowed compensation for hypocalcemia.

The patient was discharged in a satisfactory condition on Day 14 after surgery with prescription of calcium carbonate tablets 4 g per day, alfa-calcidol capsules 2 μg per day, followed by dose adjustment by an endocrinologist according to the blood calcium level. In order to correct vitamin D deficiency, calciferol was prescribed at 7,000 IU per day for 8 weeks, followed by transfer to maintenance dose of 2,000 IU per day.

The patient was examined 6 months after the surgery: she has no complaints, receives substitution therapy including calcium carbonate tablets up to 3 g per day, alfa-calcidol capsules 2 μg per day, colecalciferol 2,000 IU per day.

## DISCUSSION

The detection rate of PHPT in Russia is growing every year [1], however, insufficient alertness of primary care doctors and outpatient endocrinologists regarding the symptoms of PHPT continues to cause untimely diagnosis and treatment of the disease in a complicated form, which is illustrated by the above clinical observation. The patient was followed up for a long time by the district physician and endocrinologist at the place of residence, presented complaints specific to PHPT, however, the diagnosis was established only by an oncologist when the disease was complicated by fibrocystic osteitis and maxilla brown tumor.

Fibrocystic osteitis with development of brown tumors is the final stage of bone remodeling with prolonged PHPT [11]. Brown tumors is a giant cell reparative granuloma in which osteoclastic demineralization predominates over the mineralization of cancellous bone [6, 11]. Under the influence of osteoclasts, the hypervascular demineralized stroma undergoes hemorrhagic degeneration with hemosiderin deposition which gives it a brown color [7]. As a rule, the process involves long tubular, flat and small bones of the wrist, ribs, clavicle, pelvic bones and skull [6], and mandibular bone is affected 2-fold more often than maxilla [12]. Bone lesions and giant thyroid adenoma increase the risk of postoperative hypocalcemia and hungry bone syndrome [13].

The clinical situation described by us should be differentiated from the hereditary form of the disease — hyperparathyroidism syndrome with jaw tumor (HPT–JT). HPT–JT syndrome is a rare autosomal dominant disease characterized by PHPT development, ossifying fibroids of mandibular bone and maxilla, cystic and neoplastic kidney lesions as well as hyperplastic and neoplastic uterine lesions [6, 14]. This syndrome is based on germline mutations in the cell division cycle protein 73 homolog (CDC73) tumor suppressor gene which is located on the long arm of chromosome 1 (1q31.2) and encodes the nuclear protein parafibromin. More than 80% of CDC73 mutations are nonsense mutations that lead to a complete decrease in parafibromin expression in tissue cells associated with HPT–JT [15]. According to the consensus report of the European Society of Endocrine Surgeons (ESES) on hereditary forms of hyperparathyroidism, genetic screening for CDC73 gene mutations is indicated in the presence of signs of familial hyperparathyroidism: the onset of the disease at a young age (<40 years old), multiple PTG lesions, cystic/atypical/malignant PTG lesions or in the presence of ossifying fibroma of the jaw, tumor of the kidneys or uterus [16]. In the described clinical case, the patient had clinical signs of HPT–JT syndrome, however, due to the late onset of the disease, the absence of a burdened family history, multiple PTG lesions, and kidney and uterine damage, molecular genetic study of the CDC73 gene was not carried out.

The clinical case presented by us should also be differentiated with PTG carcinoma. It has been established that in the case of a combination of severe hypercalcemia (ionized calcium level > 1.6 mmol/l), blood iPTH level > 600 pg/ml, and volume of lesion > 6 cm3, a thyroid tumor should be considered suspicious for a malignant neoplasm [17]. According to this criterion, the situation described by us really had a high risk of malignant neoplasm of the PTG, despite the absence of signs of local invasion and distant metastases according to the examination data. In the above case, the expansion of the surgery volume up to removal of the right upper thyroid gland as enblock with the right lobe of the thyroid gland was selected taking into account the intimate relationship of anatomical structures and a high risk of malignancy.

A complicated course of PHPT with the development of severe bone lesions and fibrocystic osteitis is an indication for surgical treatment. Removal of the PTG adenoma is the first stage in the treatment of fibrocystic osteitis which leads to IPTH dcrease, a gradual increase in bone mineral density with suppression of fibrocystic changes, increased physical activity and reduced risk of fractures [18].

Expecting severe hypocalcemia in the postoperative period, intravenous administration of calcium gluconate solution was prescribed from Day 1 after surgery followed by taking vitamin D and calcium preparations that allowed rapid compensation for this condition. Postoperative hypocalcemia was caused by the development of hungry bone syndrome as well as prolonged severe hypercalcemia and suppression of normal PTG by active adenoma. Hungry bone syndrome is observed in 13% of patients who underwent parathyroidectomy for PHPT. It is based on a sharp decrease in bone resorption, activation of fibroblasts with the formation of bone tissue. It is manifested by prolonged hypocalcemia with the development of clinical symptoms (paresthesia and seizure activity) [13]. Risk factors for the development of this syndrome include skeletal damage with severe osteoporosis, giantic size of the PTG adenoma, simultaneous surgery on the PTG and thyroid gland, and higher preoperative levels of iPTH [19, 20]. In our patient, severe damage to the skeleton with the development of fibrocystic osteitis played a major role in the development of postoperative hypocalcemia. Unfortunately, due to the urgent indications for surgery, osteodensitometry was not performed in the patient.

## CONCLUSION

Despite the well–studied role of the PTG in the regulation of calcium–phosphorus metabolism and maintenance of bone mineral density, the alertness of primary care physicians including outpatient endocrinologists regarding PHPT symptoms is still insufficient. In addition, this nosological entity should be borne in mind for all unexplained tumor lesions of the jaw. With such a combination of complications, differential diagnosis should be carried out with the hereditary form of PHPT and PTG cancer. The presented case shows the result of late diagnosis — the patient practically ceased to move independently, there was a high risk of hypercalcemic crisis.
